# Significance of Systemic Scleroderma-Specific Autoantibodies in Idiopathic Interstitial Pneumonia

**DOI:** 10.7759/cureus.66986

**Published:** 2024-08-16

**Authors:** Yu Murakami, Hiroki Wakabayashi, Kaichi Kaneko, Kenta Takashima, Atsuhito Saiki, Yasuo Matuzawa

**Affiliations:** 1 Division of Diabetes, Metabolism and Endocrinology, Department of Internal Medicine, Toho University Graduate School of Medicine, Ōta City, JPN; 2 Division of Respiratory Medicine, Department of Internal Medicine, Toho University Sakura Medical Center, Sakura, JPN; 3 Division of Rheumatology, Department of Internal Medicine, Toho University Sakura Medical Center, Sakura, JPN

**Keywords:** anti-fibrillarin antibody, systemic scleroderma-specific autoantibody, systemic scleroderma, idiopathic interstitial pneumonia, interstitial lung disease

## Abstract

Objective

Patients with idiopathic interstitial pneumonia (IIP) often test positive for systemic scleroderma-specific autoantibodies (SSc-Ab), even if they do not meet the diagnostic criteria for systemic scleroderma (SSc). However, the significance of SSc-Ab in IIP is unknown.

Methods

We retrospectively studied the medical records of all patients suspected of interstitial lung disease (ILD) who visited our center between January 2016 and December 2021. We evaluated the association between SSc-Ab subtypes and clinical characteristics, prognosis, and incidence of acute exacerbation (AE) of IIP. Among 571 patients suspected of having IIP and SSc-Ab measured, we excluded cases with clear causes of ILD or those diagnosed with other diseases and analyzed 386 cases diagnosed as IIP.

Results

Among 386 IIP patients, 48 were SSc-Ab positive (platelet-derived growth factor receptor (PDGFR) in 0, Th/To in 10, anti-nucleolar organizer region 90 antibodies* (*NOR90) in 12, fibrillarin in five, RP155 in 14, RP11 in three, CENP A in seven, CENP B in 10, and Scl-70 in six). There was no significant difference in survival rate or incidence of AE between patients with or without SSc-Ab. Multivariate logistic regression analysis showed that age and malignancy were significant risk factors for death, whereas age, male sex, and anti-fibrillarin antibodies were significant risk factors for AE of IIP.

Conclusion

None of the SSc-Abs were associated with the risk of mortality, and anti-fibrillarin antibodies, along with age and male sex may contribute to the risk of AE of IIP, predicting severe lung involvement and warranting multidisciplinary treatment and careful follow-up.

## Introduction

Idiopathic interstitial pneumonia (IIP) is a general term for interstitial lung disease (ILD) with no identifiable cause [1]. IIP diagnosis requires exclusion of ILD due to autoimmune disease, and the official statement of the American Thoracic Society/European Respiratory Society/Japanese Respiratory Society/Latin American Thoracic Association (ATS/ERS/JRS/ALAT) recommends screening for autoantibodies [2]. However, there are ILDs that do not meet the diagnostic criteria for connective tissue disease (CTD) but have symptoms or autoantibodies associated with CTD. These include undifferentiated CTD (UCTD), lung-dominant CTD (LD-CTD), and autoimmune-featured ILD (AIF-ILD), each with criteria proposed by individual researchers. In 2015, ATS/ERS proposed the concept of interstitial pneumonia with autoimmune features (IPAF) as a term that encompasses all of the above. IPAF classification criteria consist of clinical, serological, and morphological domains, at least two of which must be met [3].

Systemic scleroderma-specific autoantibodies (SSc-Ab) correlate with stage and prognosis in patients with systemic scleroderma (SSc) and are useful in treatment and predicting prognosis [4]. The American College of Rheumatology/European League Against Rheumatism (ACR/EULAR) SSc classification criteria include anti-Scl-70, anti-centromere, and anti-RNA polymerase III antibodies, which are important for diagnosis. It is estimated that about 60-80% of patients with SSc have anti-centromere, anti-Scl-70, or anti-RNA polymerase III antibodies [5]. Anti-Scl-70 antibodies are a risk factor for the development and progression of ILD in patients with SSc and are a factor for poor prognosis in patients with SSc [4]. Diagnostic criteria for IPAF include anti-Scl-70 antibodies for SSc-Ab. Still, some patients who are positive for SSc-Ab but do not meet the clinical domain or who are positive for SSc-Ab are not included in the diagnostic criteria for IPAF. Although these patients who do not meet the diagnosis of IPAF are also diagnosed with IIP, the clinical significance of SSc-Ab, including these patient populations, has not been established. Few reports examine the mortality, acute exacerbation (AE), and clinical characteristics of SSc-Ab-positive IIP. Therefore, we focused on the significance of SSc-Ab in patients with IIP, including those who did not meet the diagnostic criteria for CTD or IPAF. We designed this retrospective observational study to determine the association of each SSc-Ab subtype with clinical context, prognosis, and AEs of IIP.

## Materials and methods

This was a retrospective observational study conducted on an opt-out basis. The medical records of all patients suspected with ILD who visited Toho University Sakura Medical Center, Sakura, Chiba, Japan, between January 2016 and December 2021 were retrospectively analyzed. This study was conducted in accordance with the ethical provisions of the Helsinki Declaration and approved by the Ethics Committee of Toho University Sakura Medical Center (approval number: S22011).

To analyze patients with SSc-Ab measured and diagnosed with IIP in this study, we excluded (i) patients with a known cause or underlying disease of ILD, such as CTD, drugs, infection, radiation, occupation, or environmental exposure, and (ii) patients who had a diagnosis other than ILD (e.g., heart failure). The diagnosis of SSc was made by rheumatologists based on the SSc classification criteria by EULAR/ACR, and patients meeting the diagnostic criteria for SSc were excluded [6]. CTDs other than SSc were diagnosed and ruled out by rheumatologists based on diagnostic criteria. IIPs were diagnosed by multiple pulmonologists, rheumatologists, and radiologists based on clinical findings, medical history, and chest high-resolution computed tomography (HRCT) scans in accordance with the 2013 ATS/ERS IIPs guidelines [1]. Imaging patterns were classified into usual interstitial pneumonia (UIP) patterns, nonspecific interstitial pneumonia (NSIP) patterns, organizing pneumonia (OP) patterns, diffuse alveolar damage (DAD) patterns, and other patterns. In this study, pulmonary hypertension (PH) was defined as a tricuspid regurgitation pressure gradient (TRPG) of 40 mmHg or greater on echocardiography.

SSc-Ab

The official ATS/ERS/JRS/ALAT statement recommends screening for autoantibodies in the differential evaluation of IPF. SSc-Ab was assessed using the EUROLINE Systemic Sclerosis Profile (EUROIMMUN Medizinische Labordiagnostika AG, Lübeck, Germany) at the first visit. The EUROLINE Systemic Sclerosis Profile can be detected simultaneously using line immunoassays for the following autoantibodies: platelet-derived growth factor receptor (PDGFR), Th/To, nucleolar organizing region 90 (NOR90), fibrillarin (U3RNP), RNA polymerase epitopes155 (RP155), RNA polymerase epitopes11 (RP11), centromere proteins A and B (CENP A and CENP B), DNA topoisomerase I (Scl-70). Samples were analyzed using a EUROLINE Scan flatbed scanner (EUROIMMUN Medizinische Labordiagnostika AG) to measure the signal intensity of the strips. Readings obtained with signal intensities of 0-5 (-) and 6-10 (borderline) were considered negative, while 11-25 (+), 26-50 (++), and higher (++++) were considered positive.

Definition of AE

AE of IIP was based on the diagnostic criteria proposed by the IPF International Working Group in 2016, modified in part to meet the following criteria [7]: (i) presence of findings of fibrotic ILD such as reticular shadows or honeycomb lung on HRCT, (ii) acute onset or exacerbation of dyspnea within one month, (iii) new bilateral infiltrative or frosted shadows in addition to existing findings of fibrotic ILD on HRCT, and (iv) deterioration that is not adequately explained by cardiac failure or fluid retention.

Statistical analysis

Statistical analysis was performed using IBM SPSS Statistics for Windows, Version 21.0 (Released 2012; IBM Corp, Armonk, New York, United States). Categorical variables were reported as percentages and continuous variables were reported as mean and standard deviation (SD) or median and interquartile range (IQR). When comparing the two groups, the Mann-Whitney U test was applied to numerical data, and Fisher's exact test was used for categorical data. The significance level was set at p<0.05. The Kaplan-Meier method was used to estimate the survival and incidence of AE rates between groups and a log-rank test was used to analyze statistically significant differences. The start date of the analysis was defined as the date of diagnosis of IIP. To identify predictors of death and AE, univariate and multivariate analyses were performed using logistic regression analysis with explanatory variables age, male sex, smoking history, malignancy, UIP pattern, anti-PDGFR antibody, anti-Th/To antibody, anti-NOR90 antibody, anti-fibrillarin antibody, anti-RP155 antibody, anti-RP11 antibody, anti-CENP A antibody, anti-CENP B antibody, and anti-Scl-70 antibody.

## Results

**Figure 1:**
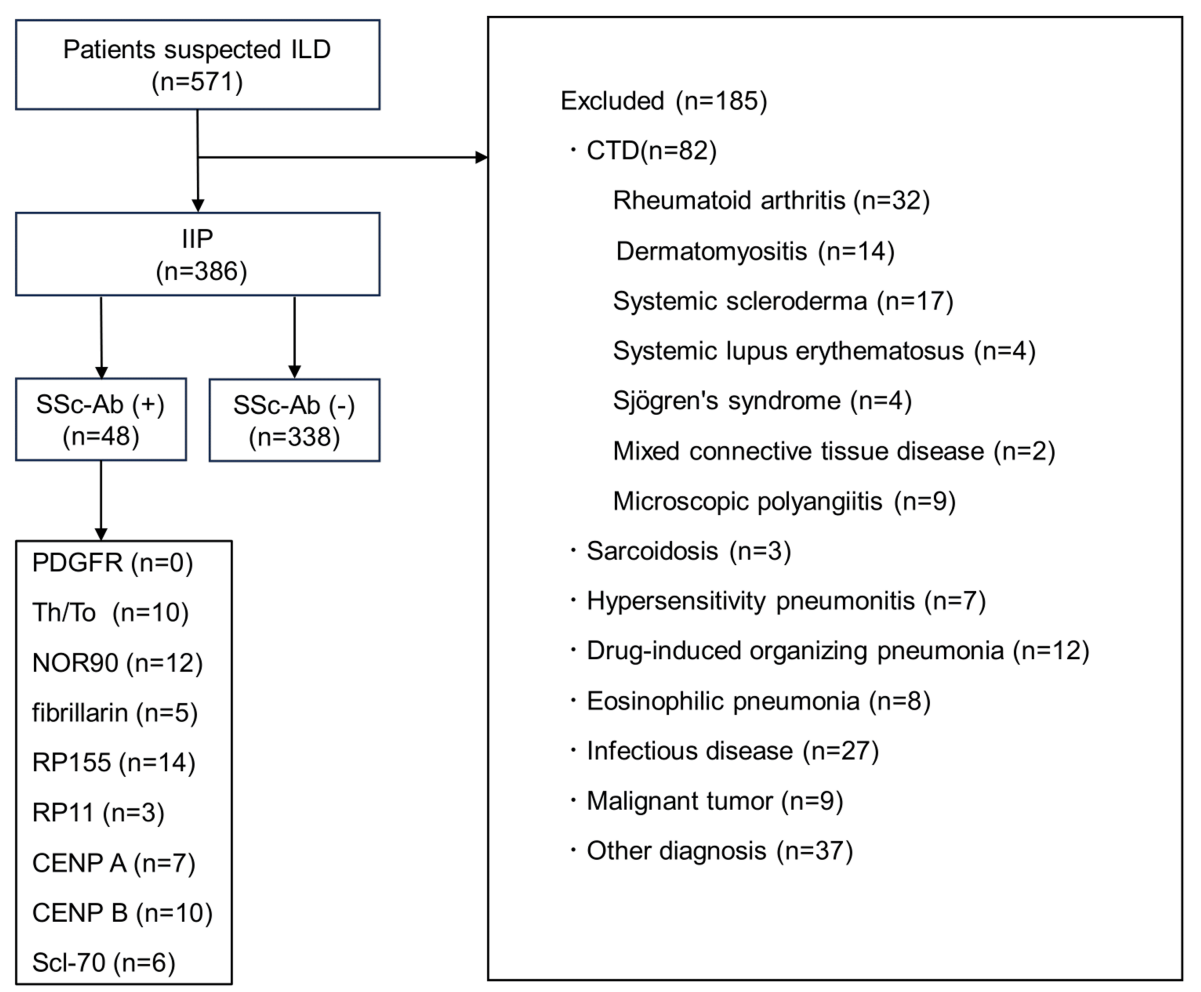
Study flowchart ILD, interstitial lung disease; IIP, idiopathic interstitial pneumonia; SSc-Ab, systemic scleroderma-specific autoantibodies; CTD, connective tissue disease.

The flow of patient selection is shown in Figure 1. Of the 571 patients with suspected ILD for whom SSc-Ab was assessed, 82 with a diagnosis of CTD within the follow-up period and 103 with other diagnoses were excluded, leaving 386 patients with a diagnosis of IIP for analysis. Of the 386 cases diagnosed with IIP, 48 were SSc-Ab positive, including anti-PDGFR antibody in zero, anti-Th/To antibody in 10, anti-NOR90 antibody in 12, anti-fibrillarin antibody in five, anti-RP155 antibody in 14, anti-RP11 antibody in three, anti-CENP A antibody in seven, anti-CENP B antibody in 10, and anti-Scl-70 antibody in six. It should be noted that of the 571 total patients with suspected ILD, 12 developed scleroderma in SSc-Ab positive patients during the follow-up period and five in SSc-Ab negative patients.

Demographic characteristics and the comparisons between SSc-Ab positive and negative groups are given in Table 1. Regarding the clinical diagnosis of IIP, there were 194 cases of IPF, 114 cases of idiopathic NSIP, 50 cases of cryptogenic organizing pneumonia (COP), 11 cases of acute interstitial pneumonia (AIP), 15 cases of idiopathic pleuroparenchymal fibroelastosis (PPFE), and two cases of unclassifiable idiopathic interstitial pneumonia. Measurement of KL-6 predicted forced vital capacity (FVC), and diagnosis of PH was performed at the time of diagnosis of IIP. Causes of death were classified as AE of IIP, chronic respiratory failure, malignancy, infection, or others. Treatments were classified as corticosteroids, immunosuppressants, or anti-fibrotic drugs. The breakdown of malignancy was as follows: 28 lung cancer, six breast cancer, six bladder cancer, one prostate cancer, one urothelial carcinoma, one esophageal cancer, 11 gastric cancer, 10 colorectal cancer, one extramammary Paget's disease, two bile duct cancer, three hepatocellular carcinomas, one malignant lymphoma, and one tongue cancer. Male sex, age, smoking history, malignancy, PH, KL-6, predicted FVC, gender-age-physiology (GAP) score, UIP pattern, OP pattern, DAD pattern, other patterns, AE of IIP, death, and breakdown of treatment did not differ significantly between the SSc-Ab positive and negative groups. NSIP patterns (%) were significantly higher in the SSc-Ab positive group than in the negative group (p = 0.008).

**Table 1:** Baseline characteristics of patients (N = 386) SSc-Ab, systemic scleroderma-specific autoantibodies; BI, Brinkman Index; PH, pulmonary hypertension; FVC, forced vital capacity; GAP score, gender-age-physiology  score; UIP, usual interstitial pneumonia; NSIP, non-specific interstitial pneumonia; OP, organizing pneumonia; DAD, diffuse alveolar damage; AE, acute exacerbation; IIP, idiopathic interstitial pneumonia. Bold values indicate p-value <0.05; ^a^ PH was defined using tricuspid regurgitant pressure gradient (TRPG) ˃40 mmHg; ^b^ Other patterns included cases with pleuroparenchymal fibroelastosis (n = 15) and unclassifiable (n = 2); ^c^ Immunosuppressants include cyclsporin, azathioprin, tacrolimus and cyclophosphamide; ^d^ Antifibrotic drugs include nintedanib and pirfenidone

Characteristics	All subjects	SSc-Ab (+)	SSc-Ab (-)	p-value
No. of patients	386	48	338	-
Male sex (%)	256 (66.3)	26 (54.2)	230 (68.0)	0.057
Age (years), mean ± SD	71.7 ± 9.0	73.1 ± 7.5	71.5 ± 9.2	0.927
Smoking history (BI > 100) (%)	226 (58.5)	26 (54.2)	200 (59.2)	0.51
Malignancy (%)	73 (18.9)	8 (16.7)	65 (19.2)	0.671
PH ^a^ (%)	57 (14.8)	6 (12.5)	51 (15.1)	0.768
KL-6 (U/mL), mean ± SD	1057.90 ± 976.66	1049.04 ± 691.33	1059.19 ± 1011.50	0.494
FVC (% predicted), mean ± SD	82.26 ± 22.01	77.29 ± 18.79	82.91 ± 22.31	0.477
GAP score, mean ± SD	3.24 ± 2.01	3.04 ± 2.27	3.27 ± 1.97	0.226
UIP pattern (%)	194 (50.3)	22 (45.8)	172 (50.9)	0.512
Non-UIP pattern				
NSIP pattern (%)	114 (29.5)	22 (45.9)	92 (27.2)	0.008
OP pattern (%)	50 (13.0)	3 (6.3)	47 (13.9)	0.139
DAD pattern (%)	11 (2.8)	1 (2.1)	10 (3.0)	0.733
Other patterns ^b^ (%)	17 (4.4)	0 (0)	17 (5.0)	0.112
AE of IIP (%)	80 (20.7)	12 (25.0)	68 (20.1)	0.435
Cause of Death				
All cause (%)	66 (17.1)	10 (20.8)	56 (16.6)	0.463
AE of IIP (%)	34 (8.8)	6 (12.5)	28 (8.3)	0.335
Chronic respiratory failure (%)	6 (1.6)	0 (0)	6 (1.8)	0.352
Malignancy (%)	9 (2.3)	1 (2.1)	8 (2.4)	0.919
Infection (%)	4 (1.0)	0 (0)	4 (1.2)	0.453
Others (%)	13 (3.4)	3 (6.3)	10 (3.0)	0.237
Treatment				
Corticosteroid (%)	155 (40.2)	18 (37.5)	137 (40.5)	0.931
Immunosuppressant ^c^ (%)	22 (5.7)	2 (4.2)	20 (5.9)	0.624
Antifibrotic drug ^d^ (%)	50 (13.0)	2 (4.2)	48 (14.2)	0.053

Prognosis

**Figure 2:**
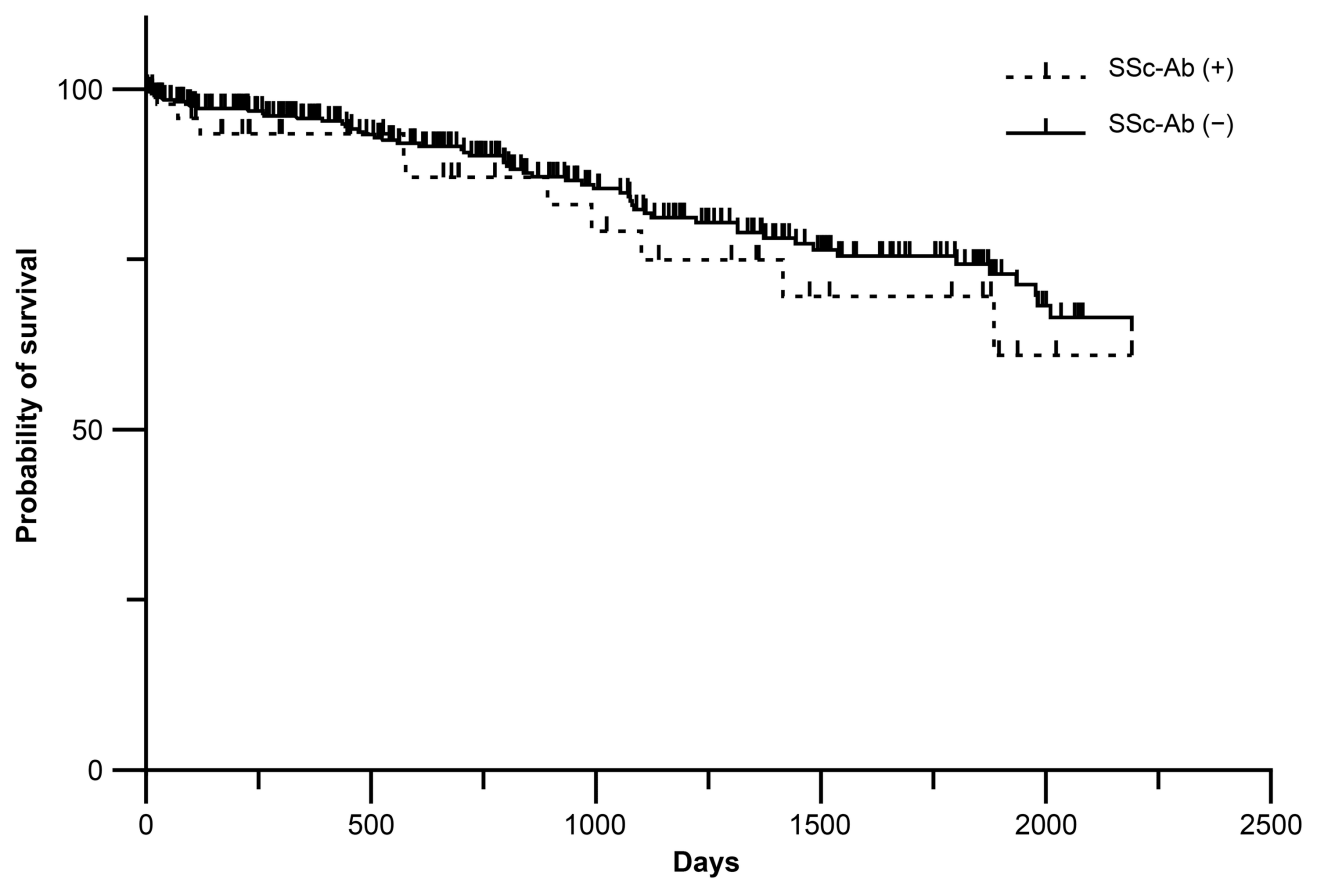
Kaplan-Meier curves for the survival in SSc-Ab positive and negative patients SSc-Ab, systemic scleroderma-specific autoantibodies.

During the median follow-up of 816.5 days (IQR 329.8-1507.3 days), 10 SSc-Ab-positive and 56 negative patients died. Distributing by antibody, deaths were of two anti-Th/To, three anti-NOR90, one anti-fibrillarin, three anti-RP155, one anti-RP11, zero anti-CENP A, one anti-CENP B, and two anti-Scl-70. Kaplan-Meier method analysis and log-rank test showed no significant difference in survival with or without SSc-Ab (p = 0.441) (Figure 2). Univariate analysis using logistic regression analysis with age, male sex, malignancy, UIP pattern, and each autoantibody as explanatory variables showed that age, malignancy, and UIP pattern were significant risk factors for death. Age and malignancy were significant risk factors for death in multivariate analysis (Table 2).

**Table 2:** Analysis of factors associated with mortality (N = 386) Bold values indicate p-value <0.05. UIP, usual interstitial pneumonia; BI, Brinkman Index

Parameter	Univariate logistic regression		Multivariate logistic regression	
	Odds Ratio (95%CI)	p-value	Odds Ratio (95%CI)	p-value
Age	1.057 (1.020–1.096)	0.002	1.064 (1.022–1.108)	0.003
Male sex	1.729 (0.942–3.175)	0.077	0.105 (0.875-4.121)	0.105
Malignancy	2.190 (1.200–3.999)	0.011	1.908 (1.014–3.591)	0.045
UIP pattern	1.790 (1.039–3.083)	0.036	1.601 (0.891–2.876)	0.115
Smoking history (BI > 100)	1.197 (0.694–2.063)	0.518	0.892 (0.441-1.806)	0.751
Th/To	1.219 (0.253–5.874)	0.805	1.396 (0.244-7.972)	0.708
NOR90	1.646 (0.433–6.249)	0.464	1.979 (0.423-9.265)	0.386
Fibrillarin	1.215 (0.134–11.051)	0.863	2.174 (0.143-33.063)	0.576
RP155	1.338 (0.363–4.933)	0.662	0.539 (0.94-3.081)	0.487
RP11	2.446 (0.219–27.378)	0.468	2.404 (0.130-44.429)	0.556
CENP A	0.000	0.999	0.000 (0)	0.999
CENP B	0.532 (0.066–4.269)	0.552	0.796 (0.55-11.418)	0.867
Scl-70	2.469 (0.443–13.766)	0.303	2.004 (0.330-12.175)	0.450

AE of IIP

**Figure 3:**
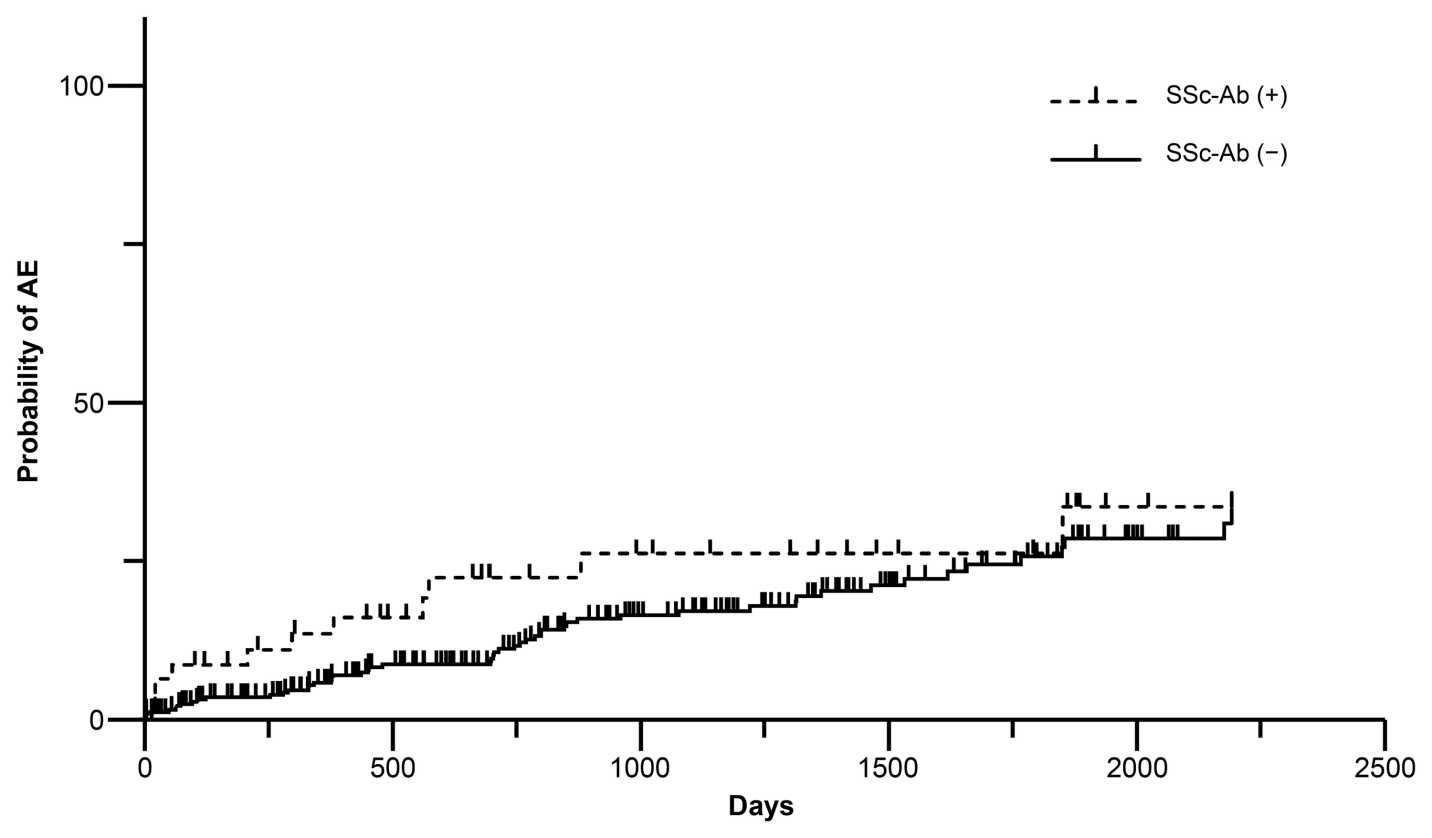
Kaplan-Meier curves for the cumulative incidence of acute exacerbation in SSc-Ab positive and negative SSc-Ab, systemic scleroderma-specific autoantibodies; AE, acute exacerbation.

During the median follow-up of 772.5 days (IQR, 290.8-1473.3 days), AE occurred in 12 SSc-Ab-positive and 68 negative patients. Distributing by antibody, two anti-Th/To, three anti-NOR90, three anti-fibrillarin, four anti-RP155, one anti-RP11, one anti-CENP A, two anti-CENP B, and one anti-Scl-70 had AE. Kaplan-Meier method analysis and the log-rank test showed no significant difference in the incidence of AE with or without SSc-Ab (p = 0.204) (Figure 3). In the univariate analysis using logistic regression analysis with age, male sex, malignancy, UIP pattern, and each autoantibody as explanatory variables, age and male sex were significant risk factors for AE of IIP. In the multivariate analysis, age, male sex, and anti-fibrillarin antibody were significant risk factors for AE of IIP (Table 3).

**Table 3:** Analysis of factors associated with acute exacerbation of idiopathic interstitial pneumonia (N = 386) Bold values indicate p-value <0.05. UIP, usual interstitial pneumonia; BI, Brinkman Index

Parameter	Univariate logistic regression		Multivariate logistic regression	
	Odds ratio (95%CI)	p-value	Odds ratio (95%CI)	p-value
Age (years)	1.035 (1.003–1.067)	0.030	1.046 (1.011–1.082)	0.009
Male sex	1.989 (1.120–3.530)	0.019	2.710 (1.289–5.698)	0.009
Malignancy	0.987 (0.526–1.853)	0.967	0.968 (0.501-1.869)	0.923
UIP pattern	0.674 (0.410–1.108)	0.120	0.551 (0.323–0.941)	0.029
Smoking history (BI > 100)	1.316 (0.791–2.190)	0.290	1.066 (0.557-2.042)	0.846
Th/To	0.955 (0.199–4.588)	0.954	0.765 (0.111-5.267)	0.786
NOR90	1.286 (0.340–4.864)	0.711	1.722 (0.392-7.554)	0.471
Fibrillarin	5.922 (0.972–36.063)	0.054	12.945 (1.397–119.971)	0.024
RP155	1.558 (0.476–5.104)	0.464	0.578 (0.115-2.902)	0.505
RP11	1.924 (0.172–21.491)	0.595	1.208 (0.079-18.583)	0.892
CENP A	0.633 (0.075–5.334)	0.674	0.563 (0.043-7.334)	0.661
CENP B	0.955 (0.199–4.588)	0.954	1.502 (0.201-11.220)	0.692
Scl-70	0.762 (0.088–6.616)	0.805	0.704 (0.077-6.445)	0.756

Anti-fibrillarin antibody-positive patients

The results of the five anti-fibrillarin antibody-positive patients are listed (Table 4). None of the five cases were complicated by malignancy. PH was not observed in four, and one patient had missing data. CT patterns included NSIP pattern in four and UIP pattern in one patient. One patient died and AE was observed in three patients. In terms of CT patterns who had AE, two had NSIP patterns and one had UIP patterns. Three cases were complicated by other SSc-Ab.

**Table 4:** Clinical features of patients with anti-fibrillarin antibodies (N = 5) Pt, patient; BI, Brinkman Index; AE, acute exacerbation; PH, pulmonary hypertension; ND, no data; UIP, usual interstitial pneumonia; NSIP, non-specific interstitial pneumonia; IIP, idiopathic interstitial pneumonia; SSc-Ab, systemic scleroderma-specific autoantibodies. ^a^ PH was defined using tricuspid regurgitant pressure gradient (TRPG) ˃40 mmHg.

Characteristics	Pt1	Pt2	Pt3	Pt4	Pt5
Age	83	60	80	71	54
Sex	male	female	male	female	female
BI	400	1200	600	0	0
Malignancy	-	-	-	-	-
Follow-up period days	298	455	71	1362	231
Follow-up days until AE	207	-	17	56	-
PH^a^	-	-	-	-	ND
CT pattern	NSIP	NSIP	UIP	NSIP	NSIP
Death	-	-	+	-	-
AE of IIP	+	-	+	+	-
Other SSc-Ab	RP155	Th/To, NOR90, RP155	Th/To, RP155	-	-

## Discussion

This study showed two findings. First, SSc-Ab did not affect the prognosis with IIP, and second, anti-fibrillarin antibodies are a risk factor for AE of IIP.

In this study, SSc-Ab was not associated with the prognosis of IIP. There have been several reports on the significance of autoantibodies in IIP. Kang et al. reported no significant difference in survival between the autoantibody positive and negative groups in patients with IIP, and the presence of autoantibodies was a significant predictor of future CTD development [8]. Collins et al. reported no significant difference in prognosis between patients with autoantibody-positive IPF without CTD-related symptoms and patients with autoantibody-negative IPF [9]. A study examining the prognosis of patients with IPAF who met the serological domains of IPAF diagnostic criteria reported that specific autoantibodies did not affect prognosis [10]. These reports support the findings of the present study that there was no significant difference in prognosis with or without SSc-Ab in IIP and that SSc-Ab was not a prognostic factor in multivariate analysis. Furthermore, whether IIP with autoantibodies has similar patient characteristics as autoantibody-negative IIP or CTD-ILD remains unclear. There have been several reports regarding the possibility that these may present different patient profiles. Yamakawa et al. compared the clinical features and prognosis of SSc-associated interstitial pneumonia with ILD that had anti-centromere, anti-Scl-70, and anti-U1RNP antibodies but did not meet SSc diagnostic criteria. The results showed a significantly lower survival rate in the former than in the latter, as well as significant differences in HRCT findings, pathology, and baseline FVC findings, concluding that the two are distinct disease entities [11]. It has been reported that patients without symptoms of CTD and with only autoantibodies and UIP patterns in HRCT may differ from IPF patients [9]. Anti-Scl-70 antibodies are generally known as a risk factor for poor prognosis and ILD in SSc, but this was not the case in this study. Since the target population of this study was IIP patients, it was considered that their patient profile may differ from that in SSc. Whether SSc-Ab positive IIP is similar to or different from autoantibody negative IIP, SSc-ILD, or IPAF patient profiles, should be investigated in further prospective studies.

In this study, anti-fibrillarin antibodies were a risk factor for AE of IIP. Anti-fibrillarin antibodies are antibodies against the 35 kDa nucleolar riboprotein called fibrillarin [12]. It is considered an autoantibody specific for SSc [4,13-15]. Anti-fibrillarin antibodies are said to be associated with male patients, younger age of onset compared to other SSc patients, phalangeal ulcers, skeletal muscle lesions, diffuse sclerosing skin lesions, severe small bowel lesions, and PH [4,12-14,16]. Prevalence is reported to be about 5-8% of SSc patients and is more frequent in African American individuals than in Caucasian or Asian patients [12,14]. There are also various reports of association with lung lesions. Roozbeh et al. reported that anti-fibrillarin antibody-positive cases in SSc had less severe lung lesions than negative cases [16]. Similarly, anti-fibrillarin antibody-positive cases had less severe lung lesions in a study of SSc patients by Kuwana et al [17]. Several other reports indicate that anti-fibrillarin antibodies were not associated with an increased frequency of pulmonary fibrosis or ILD [13,18]. Conversely, there are reports that they are associated with an increased frequency of severe lung lesions and ILDs [16,17,19].

In relation to prognosis, there are reports that patients with SSc with positive anti-fibrillarin antibodies did not have a lower survival rate compared to patients with negative SSc [13,20]. However, previous studies have reported that anti-fibrillarin antibodies are associated with poor prognosis [19,21]. Aggarwal et al. reported in a single-center observational study that the anti-fibrillarin antibody-positive group had a shorter cumulative survival from SSc diagnosis, with PH being the most common cause of death [21]. Carolina et al. reported a strong association between anti-fibrillarin antibodies and shorter survival in a cohort study of SSc patients [12]. In this report, PH and gastrointestinal lesions are mentioned as possibly being associated with disease-related mortality. These reports were based on SSc patients, and there have been no previous reports on the prevalence and clinical characteristics of anti-fibrillarin antibody-positive IIP patients; we found that anti-fibrillarin antibody positivity is associated with AE in IIP patients. Although the mechanism of induction of anti-fibrillarin antibodies in SSc is not clear, Yang et al. speculated that fibroblast activation, proliferation, and collagen production in SSc may be associated with increased fibrillarin expression and that fibrillarin exposure to the immune system in a person with genetic predisposition may lead to autoimmunity [22]. SSc causes a marked increase in collagen and other extracellular matrix proteins in various tissues, including the dermis, esophagus, and lungs [23]. Therefore, the fibrillarin-induced increase in collagen could lead to severe lung lesions if it also occurs in lung tissue. Further investigation is needed to determine the biological mechanism by which anti-fibrillarin antibodies are involved in ILD. Additionally, three of the five anti-fibrillarin antibody-positive patients in this study had complications of other antibodies. While anti-fibrillarin antibodies are believed to be exclusive of other SSc-Abs, there have been reports of cases of complications with other antibodies [13,24,25]. It is desirable to accumulate cases of patients who test positive for multiple antibodies simultaneously and to elucidate the clinical picture of these cases. In addition, in this study, it was difficult to determine which autoantibody was involved in ILD in cases with multiple autoantibodies. Furthermore, autoantibodies other than SSc-Ab may be involved in ILD. It is an important consideration that other autoantibodies may be involved in death and AE. Future analyses that consider the presence of other autoantibodies and those that use cases with autoantibodies but without ILD as a control group are desirable.

Risk factors for mortality in IPF have been identified as age, male sex, lower predicted FVC, extensive ground-glass shadows or fibrosis lesions in HRCT, and malignancy. Risk factors for AE in IPF have been shown to include low FVC, concomitant PH, high KL-6, extensive ground-glass shadows or fibrotic lesions in HRCT, and a history of previous AE [26-28]. The results in this study were similar to those for death and risk factors for AE in IPF concerning age and malignancy as prognostic factors and age and male sex as risk factors for AE. Histological or radiological UIP patterns have been reported to be a risk factor for AE [29]. However, in this study, the analysis of AE of the UIP pattern showed that it was significantly less likely to cause AEs. The use of antifibrotic drugs, etc, may have influenced the frequency of AE. On the other hand, in the prognostic analysis, although there was no significant difference, the UIP pattern tended to have a worse prognosis. We speculate that these results may be because non-UIP patterns tend to respond well to treatment with corticosteroids and other medications and have a better prognosis.

There are several limitations to this study. First, this was a retrospective study using data from medical records and involved a relatively small number of patients at a single institution. Second, a longer follow-up period could have resulted in the development of CTDs such as SSc. Third, out of 386 IIP patients, only five cases were positive for anti-fibrillarin antibodies. Although anti-fibrillarin antibodies were extracted as a risk factor for AE in multivariate analysis, these results need to be interpreted with caution. We think that further investigation is needed using prospective studies to examine the relationship of SSc-Ab to prognosis and AE and to include autoantibodies other than SSc-Ab.

## Conclusions

Analysis of the clinical characteristics of each SSc-Ab subtype in patients with IIP revealed the following two findings: First, none of the SSc-Abs were associated with the risk of mortality. Second, anti-fibrillarin antibodies, along with age and male sex may contribute to the risk of AE of IIP. Anti-fibrillarin antibodies predict severe lung involvement and could provide a rationale for considering multidisciplinary treatment and careful follow-up. Further prospective studies are needed to elucidate treatment management, prognosis, and the prediction of AEs in patients with anti-fibrillarin antibody-positive IIP.
